# Provider-Initiated HIV Testing and Counselling for Children

**DOI:** 10.1371/journal.pmed.1001650

**Published:** 2014-05-27

**Authors:** Mary-Ann Davies, Emma Kalk

**Affiliations:** School of Public Health and Family Medicine, University of Cape Town, Cape Town, South Africa

## Abstract

Mary-Ann Davies and Emma Kalk reflect on recent research by Rashida Ferrand and colleagues into barriers to provider-initiated HIV testing for older children in Zimbabwe.

*Please see later in the article for the Editors' Summary*

**Linked Research Article**

This Perspective discusses the following new study published in *PLOS Medicine*:

Kranzer K, Meghji J, Bandason T, Dauya E, Mungofa S, et al. (2014) Barriers to Provider-Initiated Testing and Counselling for Children in a High HIV Prevalence Setting: A Mixed Methods Study. PLoS Med 11(5): e1001649. doi:10.1371/journal.pmed.1001649

Rashida Ferrand and colleagues combine quantitative and qualitative methods to investigate HIV prevalence among older children receiving primary care in Harare, Zimbabwe, and reasons why providers did not pursue testing.

Prevention of mother-to-child transmission (PMTCT) interventions have significantly reduced vertical HIV transmission but coverage is uneven and substantial gaps remain [1]. The success of PMTCT programs means that most ongoing vertical transmission is likely to be postnatal from mothers not diagnosed antenatally [2]. There is also high post-partum attrition from PMTCT programs especially for infant HIV testing after breastfeeding cessation [3],[4]. Evidence that 50% of HIV-infected infants die before their second birthday and that early antiretroviral treatment (ART) significantly reduces morbidity and mortality [5], has led to an appropriate focus on early infant diagnosis (EID), with less emphasis on later testing. However, one-third of vertically infected children may be “slow progressors,” particularly if infected postnatally [6],[7]. These children may present late, often with profound immune suppression and end-organ damage [8],[9].

In this week's *PLOS Medicine*, Rashida Ferrand and colleagues [10] highlight the substantial burden of HIV infection among older children and the barriers to HIV testing in this age group. Previous studies among older children in Zimbabwe and South Africa found HIV prevalence ranging from 3% to 15% depending on the age group and study population [11],[12]. Most older children identified as infected have prior missed opportunities for earlier diagnosis, e.g., a known HIV-infected parent or sibling, previous tuberculosis treatment, hospital admission, or repeated presentation with minor infections [13]–[15]. In 2007, WHO therefore recommended routine testing of inpatients and outpatients in endemic areas [16]. Such routine testing is feasible and acceptable, although implementation and uptake are not universal [11],[12].

## Barriers to Testing Beyond Infancy

Barriers to HIV testing of older children and adolescents are multifactorial, operating at the level of the individual, health care facility, community, and national legal framework. There is perceived lack of importance of HIV testing of older children (by caregivers, health care workers [HCWs], policy-makers, and children themselves). Common misconceptions include the belief that HIV testing is only required for symptomatic children [11]–[14]; or that perinatally infected children do not survive into late childhood. More subtle symptoms are often not considered suggestive of HIV, with children reported as “asymptomatic” despite having recurrent minor illnesses, skin infections, stunting, and school failure. There is lack of knowledge about the serious consequences of untreated infection in apparently asymptomatic or mildly symptomatic children.

Disclosure and stigma are frequently reported reasons for older children not being tested. Indirect disclosure of parental status, coupled with parental guilt and the belief that a child's knowledge of his/her HIV status will cause stress and exacerbate the disease are strong disincentives to testing [11],[12],[14],[17]. Caregivers want to protect their children and themselves from discrimination within the family and community. Facility-level barriers include lack of child-friendly services, negative HCW attitudes, insufficient staff and equipment, and prohibitive cost or travel distances.

## A New Study of Barriers to HIV Testing in Older Children

Ferrand and colleagues highlight the challenges of HIV testing of older children in a study of provider-initiated HIV testing and counselling (PITC) for children aged 6–15 years in routine primary health care clinics in Harare, Zimbabwe [10]. About half of nearly 3,000 children eligible for HIV testing were not tested, because of not being offered testing (approximately 25%), refusal of consent (approximately 15%), or leaving the clinic before testing could be completed (approximately 12%). HCWs recorded reasons for not offering PITC; the most important was guardians deemed unsuitable to give consent (nearly 60%). This finding indicates the substantial role of policy-level barriers to pediatric HIV testing. For those requiring guardian consent for testing (i.e., children <16 years old in Zimbabwe), the assessment by HCWs of whether a guardian is “suitable” is a key determinant of testing access, especially where children are frequently in the care of extended families due to AIDS orphanhood or migration. Orphanhood is a well-known risk for delayed HIV diagnosis [12]. In the study by Ferrand and colleagues [10], younger children, those with previous hospital admissions, persistent skin problems, and recent poor health were more likely to be tested suggesting that guardian suitability may be less strictly assessed in unwell children. In contrast, community barriers of stigma and fear of disclosure may make HCWs more stringent in their assessment of guardian suitability as they fear negative impacts of stigma on vulnerable children identified as infected. The qualitative interviews of HCWs in the study support this notion. The relationship of the guardian to the child was not recorded so the consistency and appropriateness of HCW assessment of guardian suitability cannot be determined.

Ferrand and colleagues' findings indicate that clear HIV testing policies for children and guidance on guardianship, together with training of HCWs on such policies are needed. The HCW interviews indicated confusion about testing guidelines and regulations. Even in countries such as South Africa and Lesotho where children may consent to testing independently from 12 years of age, HCWs need training to assess the capacity of children to give full informed consent [18]. Such policies and training need to be accompanied by education of HCWs and caregivers about the high HIV prevalence among older children (5.3% in this study) and the benefits of ART before severe symptoms appear. The study also identified other barriers to HIV-testing including stockouts, long waiting times, and lack of sufficient space for confidential counselling indicating that broader health system strengthening is needed to ensure good PITC coverage in older children.

In their study Ferrand and colleagues observed that 95% of children who tested positive for HIV were linked to care, in contrast to much lower proportions reported from other studies [19],[20]. The high linkage rate is likely due to the introduction of decentralized ART at each clinic offering PITC. Over 80% of children accessed care at the clinic where they were tested. One of the main reasons for poor infant ART initiation despite early infant diagnosis (EID) scale-up is that diagnostic and ART services are located separately [19]. Decentralization of ART needs to accompany testing if testing is to improve childhood morbidity and mortality. This study is a good example of the feasibility of this practice.

Ferrand and co-workers conducted their study in routine care clinics so their findings are likely generalizable and a good reflection of routine practices. Nevertheless, as HCWs had to document reasons for not testing, the proportion of patients offered PITC may be substantially higher than in other settings. The fact that >90% of infected children had a previous missed opportunity for testing indicates suboptimal pediatric PITC coverage in most routine settings.

## Implications and Future Research

The emphasis on early HIV diagnosis and ART initiation in infants and young children is appropriate and should continue. However, it needs to be accompanied by strengthening later PITC of older children to “mop up” those not identified through PMTCT follow-up and to prevent morbidity and mortality. Improving PITC for older children may be one of the first steps in strengthening of health services for older children and adolescents. Future research should focus on identifying “best practices” for PITC in older children and adolescents that are effective, feasible, and appropriate for sub-Saharan African settings.

## Abbreviations

ART: antiretroviral treatment
HCW: health care worker
PITC: provider-initiated HIV testing and counselling
PMTCT: prevention of mother-to-child transmission
